# Hepatic hilar mass in an adolescent: a rare case of hepatobiliary tuberculosis

**DOI:** 10.1186/s12879-019-3850-5

**Published:** 2019-03-04

**Authors:** Liwei Pang, Shuodong Wu, Jing Kong

**Affiliations:** Department of Biliary and Minimally Invasive Surgery, China Medical University Shengjing Hospital Shenyang, No. 36, San Hao Street, Shenyang, 110004 Liaoning China

**Keywords:** Hepatobiliary tuberculosis, Hilar, Child

## Abstract

**Background:**

Hepatobiliary tuberculosis is a rare manifestation of *Mycobacterium tuberculosis* infection, especially in younger patients. The non-specific symptoms and signs as well as the lack of definite imaging characteristics often impedes diagnosis. Definite diagnosis of tuberculosiscan be obtained through histopathological examination; conventional anti-tuberculosis drugs and surgery are the most commonly recommended treatments.

**Case presentation:**

A previously healthy 15-year-old rural adolescent male presented with a 2-month history of weight loss and fatigue. We strongly suspected a Klatskin tumor; therefore, exploratory laparotomy was performed. However, the microscopical findings revealed a granuloma consisting of epithelioid cells, caseous necrosis, and lymphocytic infiltration, indicating caseating granulomatous inflammation and yielding a final diagnosis of hepatic hilar tuberculosis.

**Conclusion:**

Hepatic hilar tuberculosis is an extremely rare case; few physicians may have actually treated a case. This report therefore aims to improve the overall understanding of lymphatic tuberculosis of the hepatic hilum.

## Background

Hepatobiliary tuberculosis is a rare type of *Mycobacterium tuberculosis* infection, especially in children and adolescents. The disease itself does not have any characteristic signs and symptoms; moreover, there are no defined characteristics in terms of imaging studies. Diagnosis of this specific condition is therefore difficult. Histopathological examination is considered the gold standard for diagnosing this condition. Once diagnosis is confirmed, the recommended treatment is conventional anti-tuberculosis drugs and, if necessary, surgical intervention.

## Case presentation

**Fig. 1:**
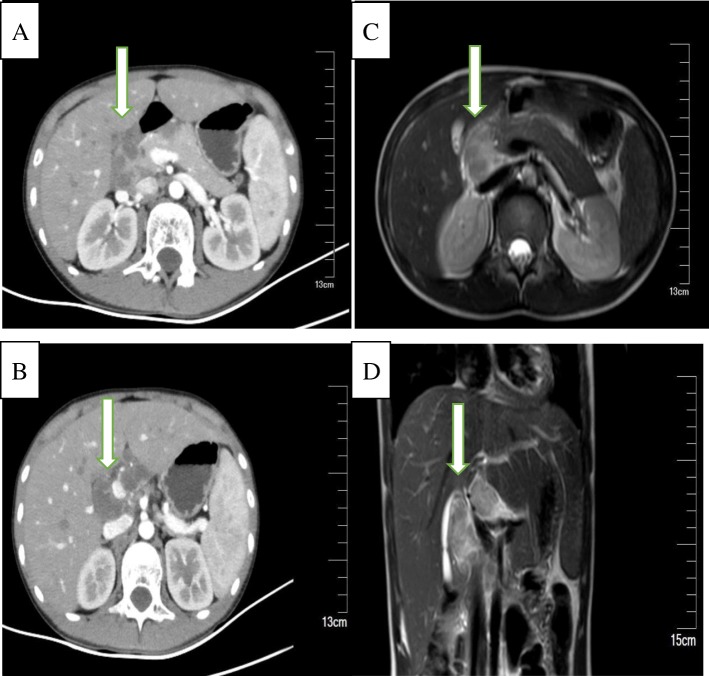
Both abdominal enhanced CT (**a**, **b**) and MRI imaging (**c**- axial, **d**- coronal) demonstrated occupied lesions in the hepatic hilum (3.0*2.7 cm), some of which are mixed, and the un-enhancing, central, low density lesion (from the hepatic hilum to the middle of the common bile duct, surrounding the hepatoduodenal ligament)with the appearance of an enhancing peripheral rim

A previously healthy 15-year-old rural adolescent male presented with the symptoms of weight loss and fatigue since 2 months. The patient reported occasional discomfort in the right upper quadrant of the abdomen, a daily nocturnal low-grade fever (37.5–38.5 °C), and a weight loss of 3.5 kg, but there was no jaundice. Physical examination yielded normal development. The patient had no tenderness or deep tenderness in the abdomen. The patient had no other typical symptoms that could point towards a specific diagnosis. There was no history of infectious diseases such as hepatitis or tuberculosis; and there was no family history of liver cancer. Enhanced computed tomography (CT) and MRI imaging revealed space-occupying lesions in the hepatic hilum (3.0*2.7 cm). Some of the lesions yielded mixed results; there was an un-enhanced, central, low-density lesion that had an enhancing peripheral rim (Fig. 1). The chest CT showed blurred nodules scattered in both lungs,the radiologist and the respiratory physician could not make a definite diagnosis it as tuberculosis. Most of the blood test results, including routine blood examination, tumor markers (AFP [0.605], CEA [0.863], CA19–9 [3.72]), thyroid hormones, liver function, and renal function were within normal limits; only the c-reactive protein level was elevated (9.08 mg/L). In addition, the results of the TPPA, HIV, TBAb, and T-spot tests were negative.

**Fig. 2:**
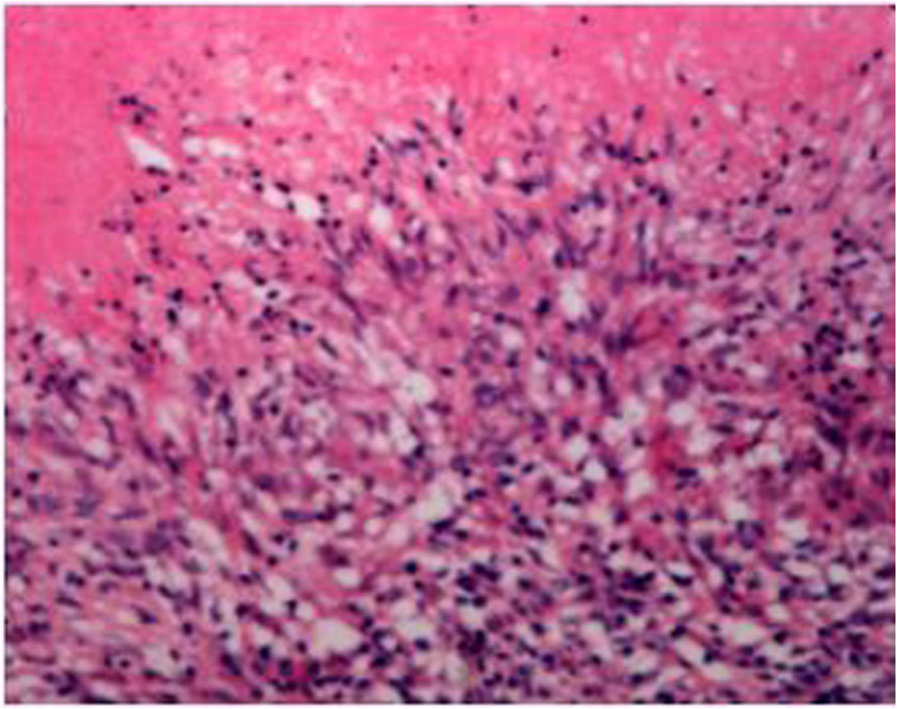
Microscopically, the findings revealed that the granuloma is composed of epithelioid cells, caseous necrosis and lymphocytesinfiltration, indicating caseating granulomatousinflammation

Based on these results, we suspected a potential diagnosis of Klatskin tumor. Exploratory laparotomy was therefore performed; diffuse small lesions were detected in the hepatic portal circulation, from the hepatic hilum to the middle of the common bile duct and surrounding the hepatoduodenal ligament. However,grass green asciteswas not observed. We resected the occupied, mixed lesions in the hepatic hilum. Histopathological examination revealed a granuloma consisting of epithelioid cells, caseous necrosis, and lymphocyte infiltration, indicating caseating granulomatous inflammation (Fig. 2). Based on these findings, the final diagnosis was extrapulmonary tuberculosis. Therefore, systemic anti-tuberculosis treatment was initiated following surgery and administered for a total of 6 months. The patient became symptom-free after two months of intensive anti-tuberculosis treatment.

## Discussion and conclusions

Lymphatic tuberculosis of the hepatic hilum, a rare form of extra-pulmonary tuberculosis, represents less than 0.1% of tuberculosis cases in adults [1]. To the best of our knowledge, few cases [2, 3] have been reported in children or adolescents.The pathogenesis of hepatic hilar tuberculosis includes direct biliary contamination from swallowed mycobacteria and extension from adjacent affected structures; hematogenous spread is rare [4]. Patients normally present with non-specific signs and symptoms such as mild fever, right upper quadrant abdominal pain, hepatomegaly, weakness, night sweats, and jaundice, which is caused by the enlarged lymph nodes compressing the common bile duct [5]. Since radiological findings are often non-diagnostic, the condition may mimic any of the common hepatic hilar lesions such as tumor, hemangiomata, and abscesses. Diagnosis is thus a challenge for clinicians and can only be confirmed through histological examination of the tissue specimens, which may yields evidence of caseous granuloma [4, 6]. Standard anti-tuberculosis therapy is an effective medical treatment regimen [7];the indications for surgery should be carefully evaluated.Based on our experience with this case and a study of the literature and our data, the indications of surgical treatment for hepatic hilar tuberculosis are: (1) isolated large tuberculoma that does not respond well to anti-tuberculosis treatment; (2) suspicion of malignant lesions; (3) patients presenting with biliary tract bleeding or acute abdomen; (4) compression of the biliary tract causing obstructive jaundice [8]. In addition, surgical excision of lesions provides relief from related symptoms and provides the tissue specimens for histological examination without the need to remove all the enlarged lymph nodes.

In conclusion, lymphatic tuberculosis of the hepatic hilum is an extremely rare condition that not all physicians may be aware of this condition. Tuberculosis should not be excluded from the differential diagnosis of atypical lesions in the hepatic hilum. Medical therapy remains the mainstay of treatment for lymphatic tuberculosis of the hepatic hilum, but if there are indicators for surgery or difficulty in diagnosis, surgery accompanied by anti-tuberculosis drug therapy could be adopted.

## Abbreviations

AFP: Alpha fetoprotein
CEA: Carcinoembryonic antigen
HIV: Human Immunodeficiency Virus
TB: Tuberculosis
TBAb: Tuberculosis antibody
TPPA: Treponema pallidum particle assay
